# Quantitative ^1^H-NMR Method for the Determination of Tadalafil in Bulk Drugs and its Tablets

**DOI:** 10.3390/molecules200712114

**Published:** 2015-07-02

**Authors:** Qingyun Yang, Hui Qiu, Wei Guo, Dongmei Wang, Xingning Zhou, Dan Xue, Jinlan Zhang, Song Wu, Yinghong Wang

**Affiliations:** Institute of Materia Medica, Chinese Academy of Medical Sciences & Peking Union Medical Colledge, Beijing 100050, China; E-Mails: yqy@imm.ac.cn (Q.Y.); qiuhui@imm.ac.cn (H.Q.); guowei@imm.ac.cn (W.G.); wangdmchina@imm.ac.cn (D.W.); xiaodou_zxn@aliyun.com (X.Z.); xuedan0628@126.com (D.X.); zhjl@imm.ac.cn (J.Z.)

**Keywords:** tadalafil, quantitative analysis, quantitative proton nuclear magnetic resonance, 2,4-dinitrotoluene

## Abstract

A simple, rapid, accurate, and selective quantitative nuclear magnetic resonance method for the determination of tadalafil in bulk drugs and its tablets was established and evaluated. Spectra were obtained in dimethylsulfoxide-*d_6_* using 2,4-dinitrotoluene as the internal standard. In this study, the method’s linearity, range, limit of quantification, stability, precision, and accuracy were validated. The results were consistent with those obtained from high-performance liquid chromatography analysis. Thus, the proposed method is a useful and practical tool for the determination of tadalafil in bulk drugs and its tablets.

## 1. Introduction

Nuclear magnetic resonance (NMR) is a unique structural tool and quantitative analytical technique for purity determination and reference material analysis [1,2]. To date, the applications of quantitative NMR (qNMR) mostly cover the identification and quantification of drugs and biological metabolites with the improvements in NMR instrumentation and technology, despite its lower sensitivity compared with mass spectrometry [3]. Unlike chromatography, qNMR does not require a high purity reference standard for the accurate quantification of the test compounds of interest. However, the technique provides several advantages, such as simple method development and easy sample preparation [4]. NMR can be considered as the primary ratio method of measurement characterized by the fact that the signal intensity is directly proportional to the number of nuclei that give rise to a specific resonance [5]. qNMR has been consequently used to study the quantities of complex samples, such as metabolites in biofluids [6,7], naturally occurring compounds in medicinal plants [8], and medicinal components in tablets [9,10], because of above factors.

**Figure 1:**
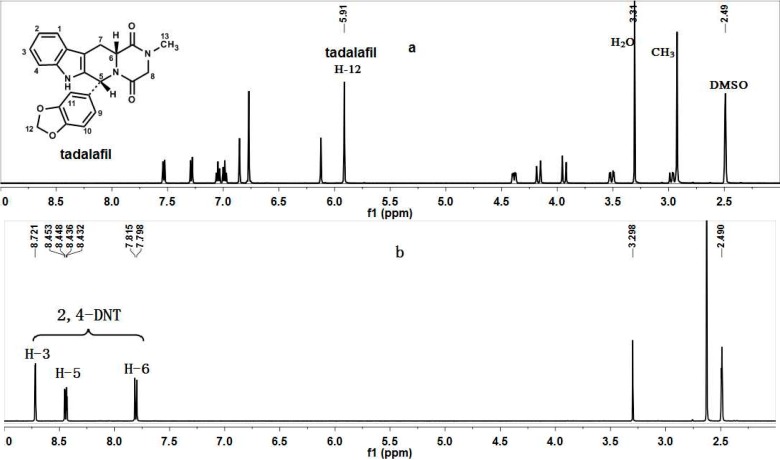

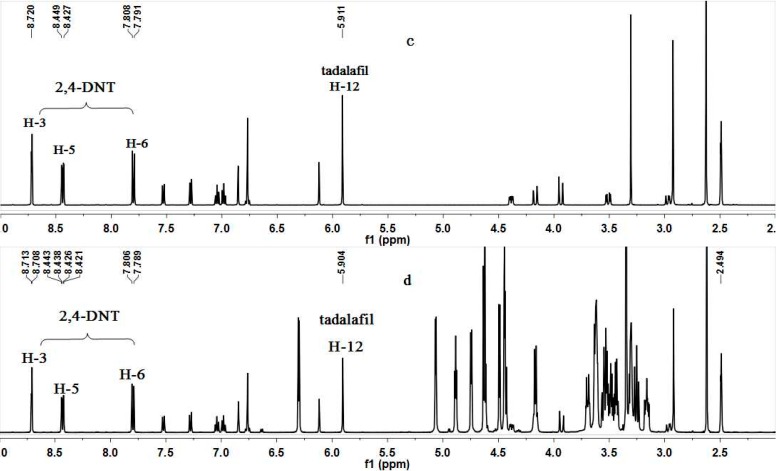
(**a**) ^1^H-NMR spectra of tadalafil in dimethylsulfoxide (DMSO). (**b**) ^1^H-NMR spectra of 2,4-dinitrotoluene (2,4-DNT) in DMSO. (**c**) ^1^H-NMR spectra of the mixture of tadalafil and 2,4-DNT in DMSO. (**d**) ^1^H-NMR spectra of the mixture of tadalafil, 2,4-DNT and tadalafil tablet powder in DMSO.

Tadalafil, (6*R*,12a*R*)-6-(1,3-benzodioxol-5-yl)-2-methyl-2,3,6,7,12,12a-hexahydropyrazino [1′,2′:1, 6]-pyrido[3,4-*b*]indole-1,4-dione, shown in Figure 1, is a drug used for treating erectile dysfunction on account of its inhibition activity on phosphodiesterase Type 5 enzyme (PDE-5) [11]. Tadalafil tablets (Cialis^®^) manufactured by Lilly del Caribe, Inc. (Carolina, PR, USA) was introduced into the European market in 2003, and brought about significant economic and social benefits. In USP (United States Pharmacopoeia) 36 [12], two different methods used to analyze the bulk drug and its tablets both rely on high-performance liquid chromatography (HPLC). Our research group has attempted to adopt various methods, such as non-aqueous titration and differential scanning calorimetry, for the quantitative determination of tadalafil. Unfortunately, all test results are unsatisfactory.

Present study provides a rapid, accurate, specific, and simple method for the assay of tadalafil using qNMR spectroscopy. The method uses 2,4-dinitrotoluene (2,4-DNT) as the internal standard for the sample [13] and dimethylsulfoxide-*d_6_* (DMSO) was used for subsequent dilutions. Good quantitative results were obtained in the determination of tadalafil as a bulk drug and in its tablets. The applicability of the proposed method was evaluated through several batches of drugs and tablets, and the results were compared with those obtained by Uniform Standards and Procedures (USP). The method was proven to be accurate, precise, selective and linear over the assessed concentration range.

## 2. Results and Discussion

### 2.1. qNMR Methods

DMSO-*d_6_* was used as the unique solvent in the NMR experiments because of its good solubilizing ability for both tadalafil and 2,4-DNT, and the latter was used as the internal standard because its NMR signals do not overlap with those of tadalafil in the ^1^H-NMR spectrum. The NMR spectra of tadalafil, 2,4-DNT, and their mixture in DMSO-*d_6_* (500 MHz) were shown in Figure 1.

The signal at *δ* 5.91 ppm (2H, s, H-12), belonging to the methylenedioxy of tadalafil in the NMR spectrum of the mixture of tadalafil and 2,4-DNT, which do not overlap with any other signals, was selected as the analytical signal for quantitative purposes. 

Figure 1 shows that the ^1^H-NMR spectrum of 2,4-DNT exhibited the characteristic signals of three aromatic protons at *δ* 8.72 (1H, d, *J* = 2.5 Hz, H-3), 8.45 (1 H, dd, *J* = 8.5, 2.5 Hz, H-5), 7.82 (1H, d, *J* = 8.5 Hz, H-6), and of methyl protons at *δ* 2.63 ppm (3H, s, CH_3_). Three well-resolved aromatic signals are suitable for the quantification because of their good resolution. However, the signals for the methyl protons were presumed inconvenient for quantification because of their long preacquisition delay caused by their long T1 relaxation time [14]. From these data, three signals at *δ* 8.72, 8.45, and 7.82 ppm were used for the quantification of tadalafil through qNMR. In particular, the signals at *δ* 8.72 ppm can be utilized for more accurate quantification because of its higher signal to noise ratio (S/N) compared with those at *δ* 8.45 and 7.82 ppm.

### 2.2. Validation

#### 2.2.1. Linearity and Range

The calibration curves showed linearity over the concentration range (*w*/*w*) of 0.47 to 3.88 for tadalafil per mg of 2,4-DNT, as shown in Table 1. The correlation coefficient obtained from the linear regression curves was 0.9999 when three aromatic protons at *δ* 8.72, 8.45, and 7.82 ppm were used as analytical signals. The results indicated that the selected method had excellent linearity over these concentration ranges. 

**Table 1:** Linearity and range of tadalafil by quantitative NMR (qNMR).

No.	*m*_std_ (mg)	*m*_x_ (mg)	*m*_x_/*m*_std_	*A*_x_/*A*_std_		
				*δ* * 8.72	*δ* 8.45	*δ* 7.82
1	5.18	2.41	0.47	0.44	0.44	0.44
2	4.83	3.45	0.71	0.67	0.67	0.67
3	5.56	5.14	0.92	0.87	0.87	0.87
4	6.46	11.72	1.81	1.68	1.68	1.67
5	5.48	14.56	2.66	2.46	2.45	2.45
6	5.00	19.40	3.88	3.64	3.64	3.64
Linear equation				y = 0.9347x − 0.0021	y = 0.9339x − 0.0030	y = 0.9344x − 0.0052
R^2^				0.9999	0.9999	0.9999

* *δ* in ppm.

#### 2.2.2. Limit of Quantification (LOQ)

The LOQ was determined according to the *S*/*N* ratio of the signal for quantification. Malz and Jancke [14] reported that a *S*/*N* ratio of at least 150 is required for the target uncertainly of 1%, and it should be greater than 150:1 for ^1^H-NMR to achieve accurate quantification. The LOQ was assessed by studying the *S*/*N* ratio and was investigated using the analyte with the minimum concentration (4.82 mg·mL^−1^) of tadalafil. In addition, the lowest *S*/*N* ratio observed was 1265.

#### 2.2.3. Precision and Stability

The results of the precision tests performed using 5 mg of sample (concentration of 0.01 mol·L^−1^) showed that the intra-day relative standard deviations (% RSD) were 0.48%, 0.48%, and 0.49% (for signals at *δ* 8.72, 8.45, and 7.82 ppm of 2,4-DNT), respectively. Therefore, the system precision was considered satisfactory. Table 2 shows that the stability was determined using the same sample at 0, 1, 2, 4, 8, and 24 h, and the % RSD values were 0.54%, 0.20%, and 0.00% for the three signals.

**Table 2:** Precision and stability of tadalafil by qNMR.

	No.	*m*_std_ (mg)	*m*_x_ (mg)	*P*_x_ (%)		
				*δ* * 8.72	*δ* 8.45	*δ* 7.82
Precision (*n* = 3)	1	4.67	5.11	99.87	100.04	100.21
	2	5.29	5.37	100.13	100.13	99.81
	3	5.13	5.73	99.51	99.51	99.17
	4	5.02	5.61	100.84	100.84	100.32
	5	5.95	5.43	99.59	99.59	99.17
	6	5.56	5.14	100.11	99.82	99.82
	Average value	/	/	100.01	99.99	99.75
	RSD%	/	/	0.48	0.48	0.49
Repeatability	1			100.71	100.71	100.19
	2			100.21	100.21	100.21
	3			99.70	100.21	100.21
	4			99.70	99.70	100.21
	5			99.70	100.21	99.70
	6			99.70	100.21	100.21
	Average value			99.95	100.21	100.12
	RSD%			0.43	0.32	0.21
Stability	0 ^#^			100.11	99.62	99.62
	1			101.10	100.11	99.62
	2			100.11	100.11	99.62
	4			99.62	100.11	99.62
	8			99.62	100.11	99.62
	24			100.11	100.11	99.62
	Average value			100.11	100.03	99.62
	RSD%			0.54	0.20	0.00

* *δ* in ppm; ^#^ Time in h.

#### 2.2.4. Recovery Tests

The proposed method, which combined solvent extraction and qNMR to determine the tadalafil content of tablets, was standardized by analyzing nine mixtures with known composition of tadalafil, tadalafil tablet powder, and 2,4-DNT. Figure 1 shows that the three signals at *δ* 8.72, 8.45, and 7.82 ppm of 2,4-DNT, and the signal at *δ* 5.91 ppm of tadalafil were well separated from the other groups. Moreover, the signals of the other tablet ingredients were minimal or only at the noise level in this zone. The signals at *δ* 8.72, 8.45, 7.82 (of 2,4-DNT), and 5.91 ppm (of tadalafil) were used for the quantification. Quantitative tadalafil was added to the tablets to investigate recovery of tadalafil from tablets, Table 3 shows the amounts used. The average recoveries of tadalafil were 100.12%, 100.71%, and 99.06%, with % RSD values 1.81%, 1.78%, and 1.40% for three signals, respectively. These results indicated that the relative proportions of tadalafil and internal standard have no influence on the method accuracy considering that tadalafil was quantitatively recovered.

**Table 3:** Recovery of tadalafil from tablets.

No.	Tadalafil					Recovery (%)		
	In Tablets (mg)	Added (mg)	Found (mg)					
			*δ* * 8.72	*δ* 8.45	*Δ* 7.82	*δ* 8.72	*δ* 8.45	*δ* 7.82
1	2.51	2.00	4.48	4.53	4.48	98.60	101.04	98.60
2	2.45	1.84	4.30	4.31	4.30	100.75	101.56	100.75
3	2.54	2.02	4.53	4.54	4.51	98.50	99.17	97.50
4	2.46	2.48	4.96	5.00	4.95	101.10	102.65	100.72
5	2.50	2.59	5.11	5.12	5.04	100.71	101.16	98.04
6	2.52	2.52	4.96	4.96	5.04	96.80	96.80	99.90
7	2.52	3.00	5.60	5.54	5.44	102.65	100.66	97.26
8	2.50	2.93	5.44	5.45	5.44	100.37	100.78	100.37
9	2.52	3.01	5.58	5.61	5.48	101.60	102.54	98.38
Average value	/	/	/	/	/	100.12	100.71	99.06
RSD%	/	/	/	/	/	1.81	1.78	1.40

* δ in ppm.

### 2.3. Sample Analysis

The established analysis method was utilized for the determination of tadalafil in bulk drugs and in its tablets compared with the conventional method using HPLC. As previously mentioned, the signals at *δ* 8.72, 8.45, and 7.82 ppm of 2,4-DNT were clearly separated from the interference signals. Therefore, the three signals were used for the quantification of tadalafil in qNMR method. The results are presented in Table 4. In all samples, no significant differences were found between the tadalafil contents determined through the proposed method compared with the conventional method. These results indicated that the accuracy of the qNMR method was comparable to that of the HPLC method. Accordingly, the qNMR method is available as an alternative method.

**Table 4:** Determination and statistical results of the qNMR and HPLC methods.

Samples	Batch No.	Specifications (mg)	qNMR Method (*n* = 6)			HPLC Method	
			*δ* *	%	RSD%	%	RSD%
Tadalafil	140501	/	8.72	100.38	1.31	99.99	0.66
			8.45	99.73	0.30		
			7.82	99.38	0.45		
	140502	/	8.72	100.06	0.19	99.90	0.94
			8.45	99.83	0.39		
			7.82	99.77	0.25		
	140503	/	8.72	99.91	0.38	100.05	1.02
			8.45	100.06	0.30		
			7.82	99.09	0.54		
Tadalafil tablets	140710	5	8.72	99.01	0.90	99.73	1.15
			8.45	99.31	0.88		
			7.82	100.22	0.98		
	C219428	5	8.72	99.10	1.33	99.23	0.84
			8.45	99.27	0.68		
			7.82	99.53	0.68		
	140725	20	8.72	99.70	2.01	100.36	1.05
			8.45	98.98	1.80		
			7.82	100.37	0.74		
	C023347	20	8.72	99.87	1.05	101.98	1.16
			8.45	100.62	1.13		
			7.82	101.54	1.06		

* *δ* in ppm.

## 3. Experimental Section 

### 3.1. Materials

The bulk of API of tadalafil (Batch No. 20140501, 20140502, and 20140503) were produced in our synthesis laboratory. Tadalafil tablets (Batch No. 20140710, 5 mg; and 2014725, 20 mg) were produced in our pharmaceutical laboratory, and commercial tablet was purchased from Lilly del Caribe, Inc., (Batch No. C219428, 5 mg; and Batch No. C023347, 20 mg). The USP tadalafil reference standard 99.9% (standard for HPLC) was purchased from USP (cat. No. 1642879; Lot. FOL003). The 95% 2,4-dinitrotoluene (2,4-DNT) (standard for qNMR) was purchased from Dr. Ehrenstorfer GmbH, German (Lot. 00903). Deuterated solvent (DMSO-*d_6_*, 99.9%) was purchased from Sigma-Aldrich (Louis, MO, USA). 

### 3.2. Instrumentation

All ^1^H-NMR spectra were obtained at 298.15 K using a Bruker Avance Spectrometer at 500.06 MHz proton frequency (AV-III-500, Burlingame, CA, USA). The qNMR experiments were performed with the following optimized parameters: pulse angle, 90°; pulse width, 15.1 μs; data points 60 K; and number of scans, 32; acquisition time (AQ), 3.172 s; spectral width (SW), 10330.578 Hz. A line-broadening factor of 0.3 Hz was applied to FIDs before Fourier transformation. The repetition delay was 60 s, which was calculated using the inversion recovery pulse program. All chemical shifts were reported in parts per million (ppm) relative to dimethylsulfoxide (DMSO-*d_6_*) at 2.49 ppm. ^1^H-NMR spectra were automatically corrected for phase and baseline distortions using TOPSPIN (version 3.0, Bruker Biospin, Spring, TX, USA). For statistical reasons, each measurement was repeated six times. The values of T1 relaxation time for targeted protons were listed as below: for 2,4-DNT, *δ* 8.72 ppm (H-3), T1 = 3.076 s; *δ* 8.45 ppm (H-5), T1 = 2.834 s; *δ* 7.82 ppm (H-6), T1 = 2.765 s; for tadalafil, *δ* 5.91 ppm (H-12), T1 = 0.986 s.

HPLC analysis was performed using a Shimadzu liquid chromatography UFLC-20ADXR equipped with a SPD-M20A spectrophotometric detector (Shimadzu Co., Kyoto, Japan). 

The analysis of tadalafil was performed using a column (zorbax SB C8, 250 mm ° 4.6 mm ID., 5 μm, Agilent, Santa Clara, CA, USA) maintained at 40 °C. The mobile phase A was a trifluoroacetic acid aqueous solution (1.0 mL trifluoroacetic acid was added to 1 L water), and mobile phase B was acetonitrile. The mobile phases A and B were mixed at a ratio of 55:45 (*v*/*v*). Flow rate was kept at 1.5 mL/min, injection volume was 20 μL, ultraviolet (UV) detection was carried out at 285 nm.

The analysis of tadalafil tablets were performed using a column (zorbax SB C8, 150 mm ° 4.6 mm ID., 5 μm, Agilent) maintained at 35 °C with acetonitrile-water-trifluoroacetic acid (35:65:0.1) (*v:v:v*) as the mobile phase. The flow rate was maintained at 1.0 mL/min, the injection volume was 10 μL, and UV detection was conducted at 285 nm.

### 3.3. Sample Preparation and Calculations

#### 3.3.1. qNMR Analysis of Tadalafil

The sample (tadalafil, about 5 mg) and internal standard (2,4-DNT, about 5 mg) were accurately weighed and transferred into a stoppered tube, and then about 0.5 mL of DMSO-*d_6_* were added. The solution was mixed using a vortex mixer until the sample and internal standard were completely dissolved. The clear solution was transferred into an analytical NMR tube, and then, the spectrum was obtained (see supplemental materials Figure S1–3 and Table S1). The purity of the analyte *P*_x_ was calculated using the following Equation (1):

(1) $$P\text{x}=\frac{A\text{x}}{A\text{std}}\frac{N\text{std}}{N\text{x}}\frac{M\text{x}}{M\text{std}}\frac{m\text{std}}{m\text{x}}\times P\text{std}\times 100\%$$

where *A*_x_ is the integral value of the signal that belongs to tadalafil; *A*_std_ is the integral value of the signal that belongs to 2,4-DNT; *N*_std_ and *N*_x_ correspond to the number of spins of 2,4-DNT (*N* = 1) and tadalafil (*N* = 2), respectively; *M*_x_ and *M*_std_ are the molecular weight of tadalafil (389.4) and 2,4-DNT (182.14), respectively; *m*_std_ and *P*_std_ are the weighted mass and the purity of 2,4-DNT, respectively; and *m*_x_ is the weighted mass of tadalafil.

#### 3.3.2. qNMR Analysis of Tadalafil in Tablets

For the assay of the tablets, 20 tablets were weighed and finely powdered. A portion of the well-mixed powder equivalent to 5 mg of tadalafil and 5 mg of internal standard were accurately weighed and transferred into a stoppered tube. The mixture was dissolved in 0.5 mL of DMSO-*d_6_* using a vortex mixer. After centrifugation, the supernatant was transferred into an analytical NMR tube, and the spectrum was obtained (see supplemental materials Figure S4). The amount of tadalafil per unit dose was calculated using the following Equation (2):

(2) $$m\text{x}=\frac{A\text{x}}{A\text{std}}\frac{N\text{std}}{N\text{x}}\frac{M\text{x}}{Mstd}\frac{m\text{std}}{m\text{powder}}\times P\text{std}\times T$$

where *m*_powder_ is the weighted mass of tablet powder sample taken for the assay; and *T* is the average tablet weight. The significance of other parameters are identical to the quantitative assay of tadalafil.

#### 3.3.3. HPLC Analysis of Tadalafil

A total of 25 mg of the substance to be examined was dissolved in 25 mL of acetonitrile and diluted to 50 mL with mobile phase A (add 1.0 mL of trifluoroacetic acid to 1 L of water). For 10.0 mL of this solution, 25 mL of acetonitrile was added and diluted to 50 mL with mobile phase A. This solution was used as the sample solution. Meanwhile, the standard solution was prepared using the same method as with USP tadalafil RS. The sample and standard solutions were subjected to HPLC analysis. The purity of tadalafil (C_22_H_19_N_3_O_4_) was calculated using the Equation (3):

(3) $$P\text{x}=\frac{A\text{x}}{A\text{s}}\frac{m\text{s}}{m\text{x}}\times P\text{s}\times 100\%$$

where *A*_x_ and *A*_s_ are the peak response from sample and standard solutions; *m*_s_ and *m*_x_ are the weighted mass of USP tadalafil RS and tadalafil substance, respectively; and *P*_s_ is the purity of USP tadalafil RS.

#### 3.3.4. HPLC Analysis of Tadalafil in Tablets

Twenty tablets were weighed and finely powdered. A portion of the well-mixed powder equivalent 5 mg of tadalafil was accurately weighed and transferred into a 100 mL volumetric flask. The flask was filled with diluent until about halfway (acetonitrile-water 1:1), and the mixture was sonicated for about 15 min to dissolve the principal component (tadalafil). The solution was then diluted with the diluent for volume and filtration. The filtrate was used as the sample solution. The standard solution was 0.25 mg·mL^−1^ USP tadalafil RS in diluent. The amount of tadalafil per unit dose was calculated using the Equation (4) below:

(4) $$m\text{x}=\frac{A\text{x}}{A\text{s}}\frac{m\text{s}}{m\text{powder}}\times P\text{std}\times T$$

where *m*_powder_ is the weighted mass of tablet powder sample obtained for the assay; and *T* is the average tablet weight.

## 4. Conclusions 

A qNMR method was developed and validated for tadalafil content determination in different batches of bulk drugs and tablets of various specifications. The method was prove to be accurate, precise, selective, and linear over the assessed concentration range. A unique aspect of the NMR spectra is the direct proportionality between the peak areas and number of protons responsible for the peak. Therefore, qNMR is an absolute quantification method with more specificity on account as no interferences were noted as with the HPLC procedures. In addition, the advantage of the qNMR method is that no authentic analyte standard is required for the analysis. Comparing the qNMR method with the HPLC approach, the contents of tadalafil in the bulk drugs and tablets were almost identical. This result indicated that the qNMR method is a useful and practical tool for the determination of tadalafil in bulk drugs and its tablets, which could be used as a complementary procedure for the HPLC method for tadalafil determination.

## Supplementary Materials

**Figure S1****:** ^1^H-NMR spectrum of tadalafil in DMSO-*d_6_* (500 MHz), **Figure S2****:** ^1^H-NMR spectrum of 2,4-DNT in DMSO-*d_6_* (500 MHz), **Figure S3****:** ^1^H-NMR spectrum of the mixture of tadalafil and 2,4-DNT in DMSO-*d_6_* (500 MHz), **Figure S4****:** ^1^H-NMR spectrum of the mixture of tadalafil, tadalafil tablet powder and 2,4-DNT (500 MHz); **Table S1****:** Chemical structure and ^1^H-NMR assignment of tadalafil. Supplementary materials can be accessed at: http://www.mdpi.com/1420-3049/20/07/12114/s1.

## References

[1] Schoenberger T. (2012). Determination of standard sample purity using the high-precision ^1^H-NMR process. Anal. Bioanal. Chem..

[2] Liu S.Y., Hu C.Q. (2007). A comparative uncertainty study of the calibration of macrolide antibiotic reference standards using quantitative nuclear magnetic resonance and mass balance methods. Anal. Chim. Acta.

[3] Simmler C., Napolitano J.G., McAlpine J.B., Chen S.N., Pauli G.F. (2014). Universal quantitative NMR analysis of complex natural samples. Curr. Opin. Biotech..

[4] Holzgrabe U., Deubner R., Schollmayer C., Waibel B. (2005). Quantitative NMR spectroscopy—Applications in drug analysis. J. Pharm. Biomed. Anal..

[5] Ohtsuki T., Sato K., Sugimoto N., Akiyama H., Kawamura Y. (2012). Absolute quantification for benzoic acid in processed foods using quantitative proton nuclear magnetic resonance spectroscopy. Talanta.

[6] Salem A.A., Mossa H.A. (2012). Method validation and determination of levofloxacin, metronidazole and sulfamethoxazole in an aqueous pharmaceutical, urine and blood plasma samples using quantitative nuclear magnetic resonance spectrometry. Talanta.

[7] Garrido R., Puyada A., Fernandez A., Gonzalez M., Ramirez U., Cardoso F., Valdes Y., Gonzalez D., Fernandez V., Verez V., Velez H. (2012). Quantitative proton nuclear magnetic resonance evaluation and total assignment of the capsular polysaccharide *Neisseria meningitides* serogroup X. J. Pharm. Biomed. Anal..

[8] Staneva J., Denkova P., Todorova M., Evstatieva L. (2011). Quantitative analysis of sesquiterpene lactones in extract of *Arnica montana* L. by ^1^H-NMR spectroscopy. J. Pharm. Biomed. Anal..

[9] Goger N.G., Parlatan H.K., Basan H., Berkkan A., Ozden T. (1999). Quantitative determination of azathioprine in tablets by ^1^H-NMR spectroscopy. J. Pharm. Biomed. Anal..

[10] Zoppi A., Linares M., Longhi M. (2005). Quantitative analysis of enalapril by ^1^H-NMR spectroscopy in tablets. J. Pharm. Biomed. Anal..

[11] Trefi S., Routaboul C., Hamieh S., Gilard V., Martino M.M., Martino R. (2008). Analysis of illegally manufactured formulations of tadalafil (Cialis) by ^1^H-NMR, 2D DOY ^1^H-NMR and Raman spectroscopy. J. Pharm. Biomed. Anal..

[12] (2013). The United States Pharmacopeia (USP).

[13] Chen Y.L., Tao L.H., Hou H., Zhang Y., Zhou X.D., Fan X.J. (2009). Quantitative determination of berberine hydrochloride by proton nuclear magnetic resonance spectroscopy with internal standard method. J. Third Mil. Med. Uni..

[14] Malz F., Jancke H. (2005). Validation of quantitative NMR. J. Pharm. Biomed. Anal..

