# Supplementary material for: Quantitative 1H-NMR Method for the Determination of Tadalafil in Bulk Drugs and its Tablets
Source: Molecules. 2015 Jul 2;20(7):12114–24. doi: 10.3390/molecules200712114 (PMC6332277; doi:10.3390/molecules200712114)
Supplement: Supplementary file 1 [file molecules-20-12114-s001.pdf]

Supplementary Materials

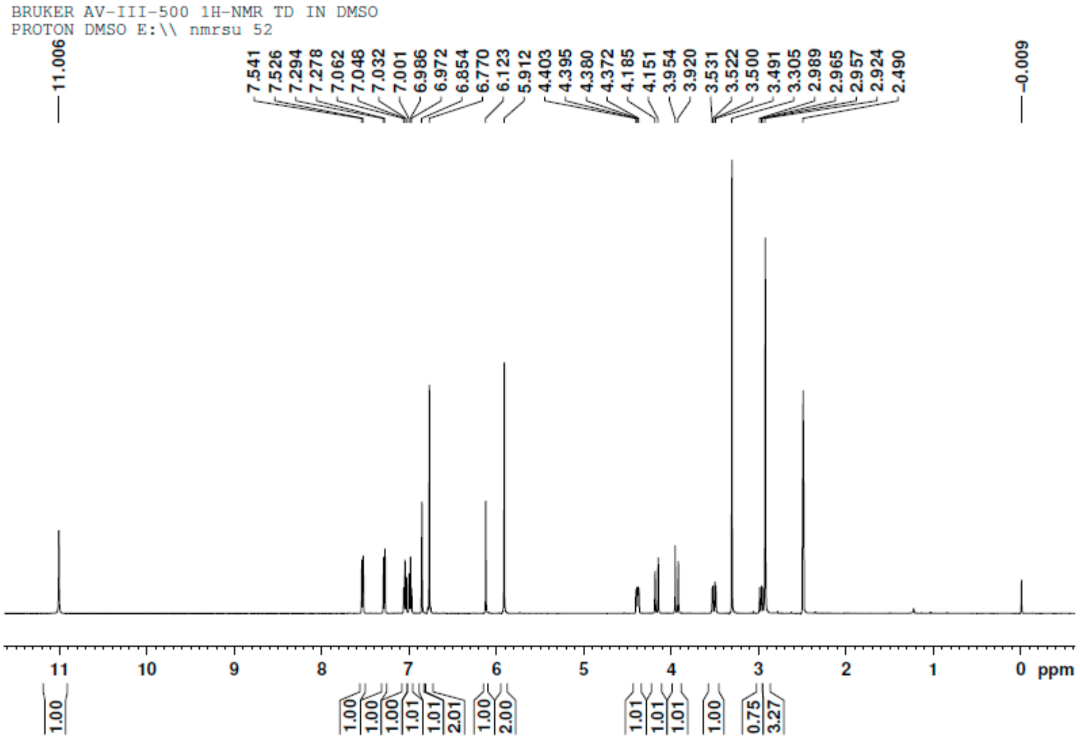

Figure S1. ^{1}H-NMR spectrum of tadalafil in DMSO-d_6 (500 MHz).

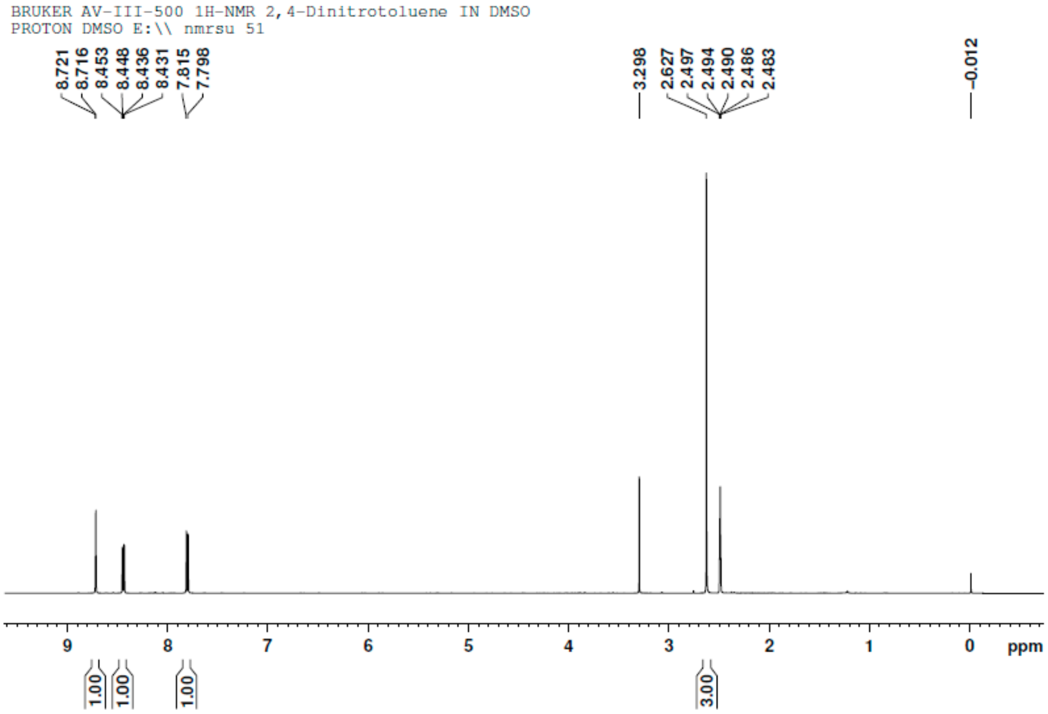

Figure S2. ^{1}H-NMR spectrum of 2,4-DNT in DMSO-d_6 (500 MHz).

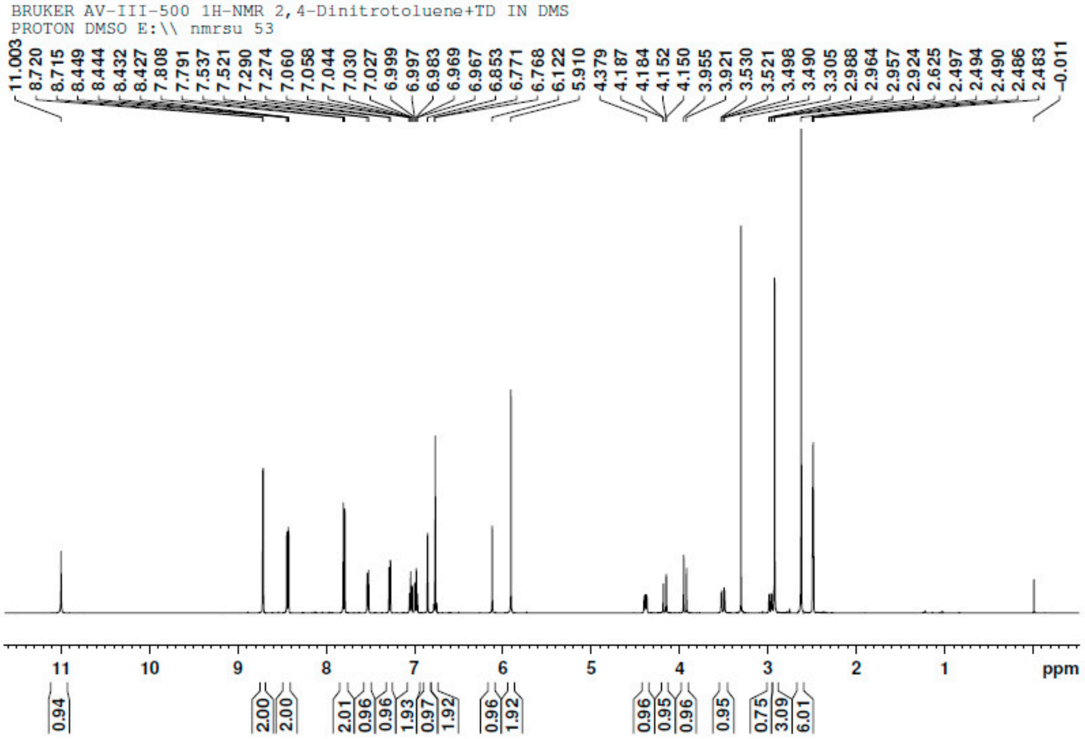

Figure S3. ^{1}H-NMR spectrum of the mixture of tadalafil and 2,4-DNT in DMSO-d_6 (500 MHz).

Table S1. Chemical structure and ^{1}H-NMR assignment of tadalafil.

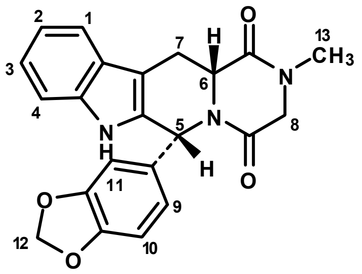

No.	δ_H	No.	δ_H
1	7.54 (1H, , d, J = 7.5Hz)	8a	4.18 (1H, d, J = 17.0Hz)
2	7.06 (1H, t, J = 7.0Hz)	8b	3.95 (1H, d, J = 17.0Hz)
3	7.00 (1H, t, J = 7.5Hz)	9	6.77 (2H, s)
4	7.29 (1H, d, J = 8.0Hz)	10	
5	6.12 (1H, s)	11	6.85 (1H, s)
6	4.40 (1H, dd, J = 11.5, 4.0Hz)	12	5.91 (2H, s)
7a	3.53 (1H, dd, J = 15.5, 4.5Hz)	13	2.,92 (3H, s)
7b	2.99 (1H, dd, J = 12.0, 4.0Hz)	NH	11.01 (1H, s)

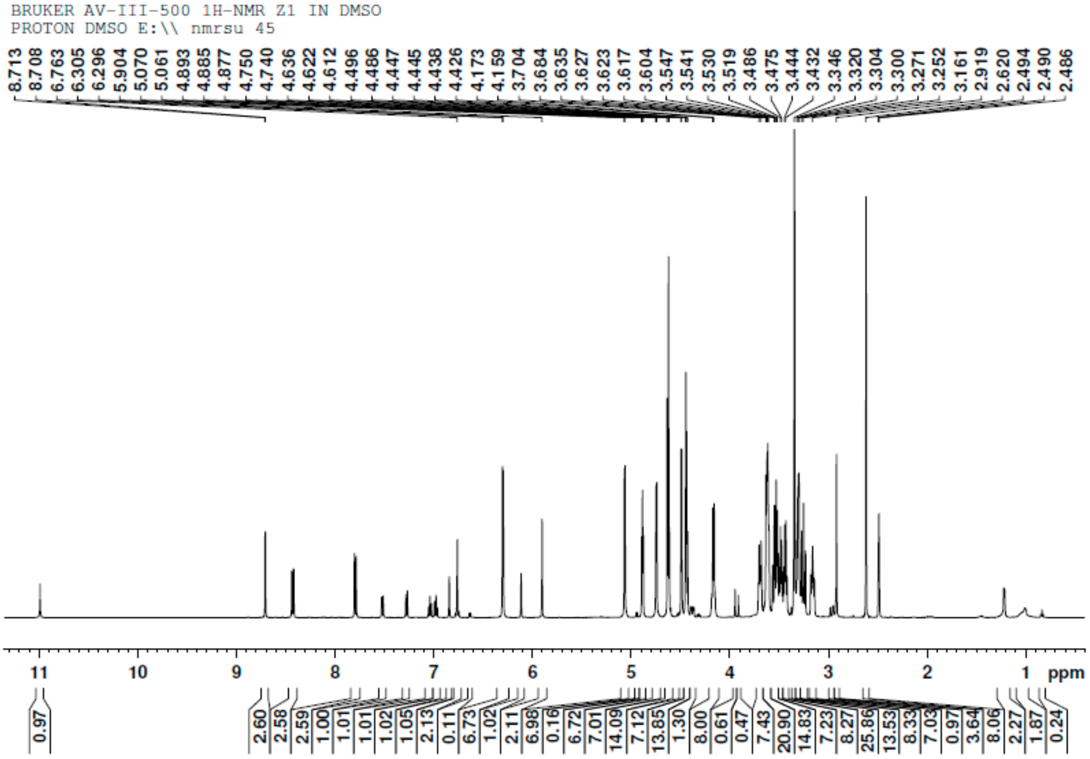

Figure S4. ^{1}H-NMR spectrum of the mixture of tadalafil, tadalafil tablet powder and 2,4-DNT (500 MHz).
